# Supplementary material for: Identification of B cell subsets based on antigen receptor sequences using deep learning
Source: Front Immunol. 2024 Mar 21;15:1342285. doi: 10.3389/fimmu.2024.1342285 (PMC10991714; doi:10.3389/fimmu.2024.1342285)
Supplement: Supplementary file 1 [file DataSheet_1.docx]

Supplementary Material


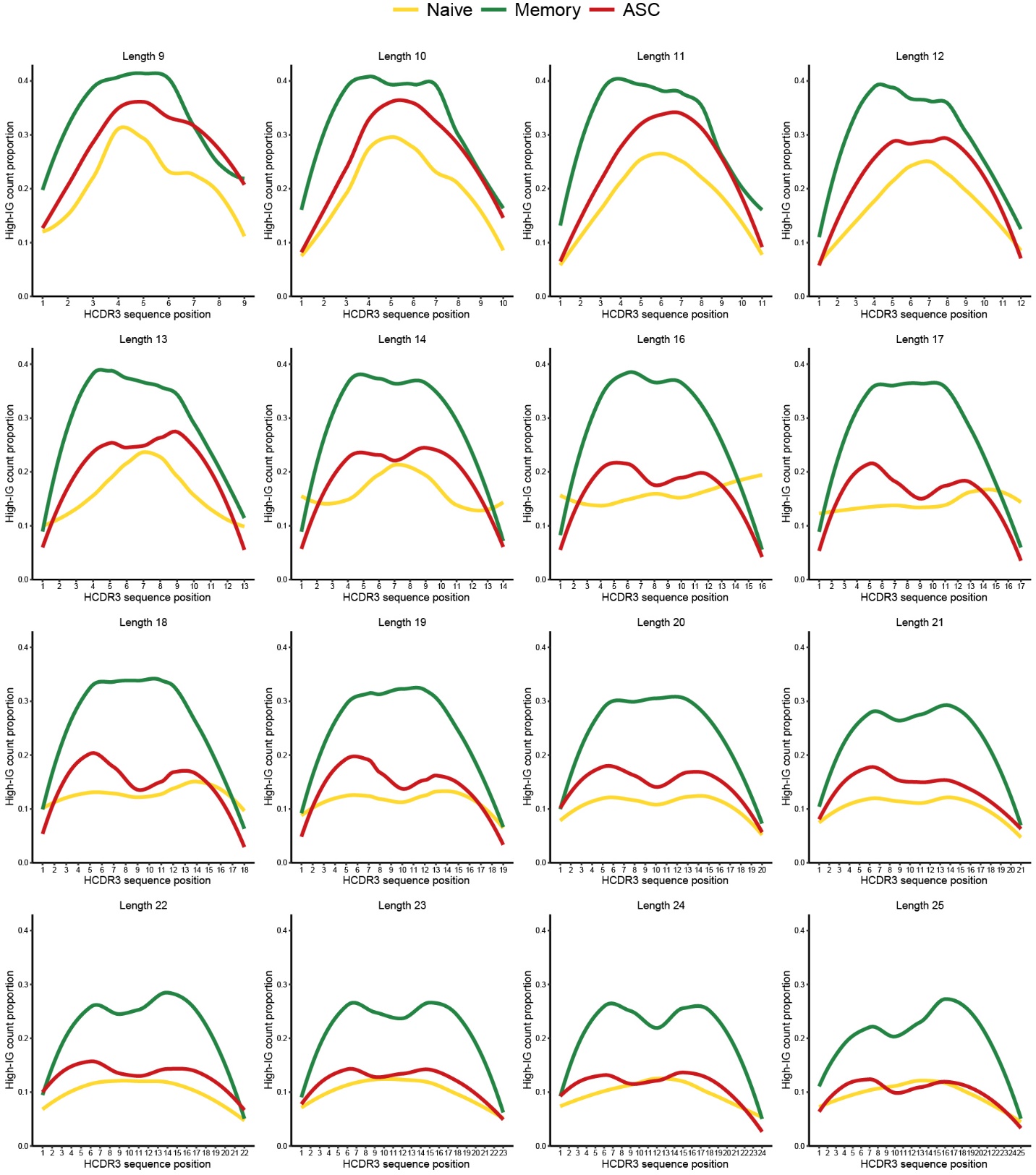


**Supplementary Figure 1.** Proportion of high-IG HCDR3 residues with regard to position. All of the HCDR3 sequence lengths from 8 to 25 except 15 (which is shown in the main figure) are plotted.


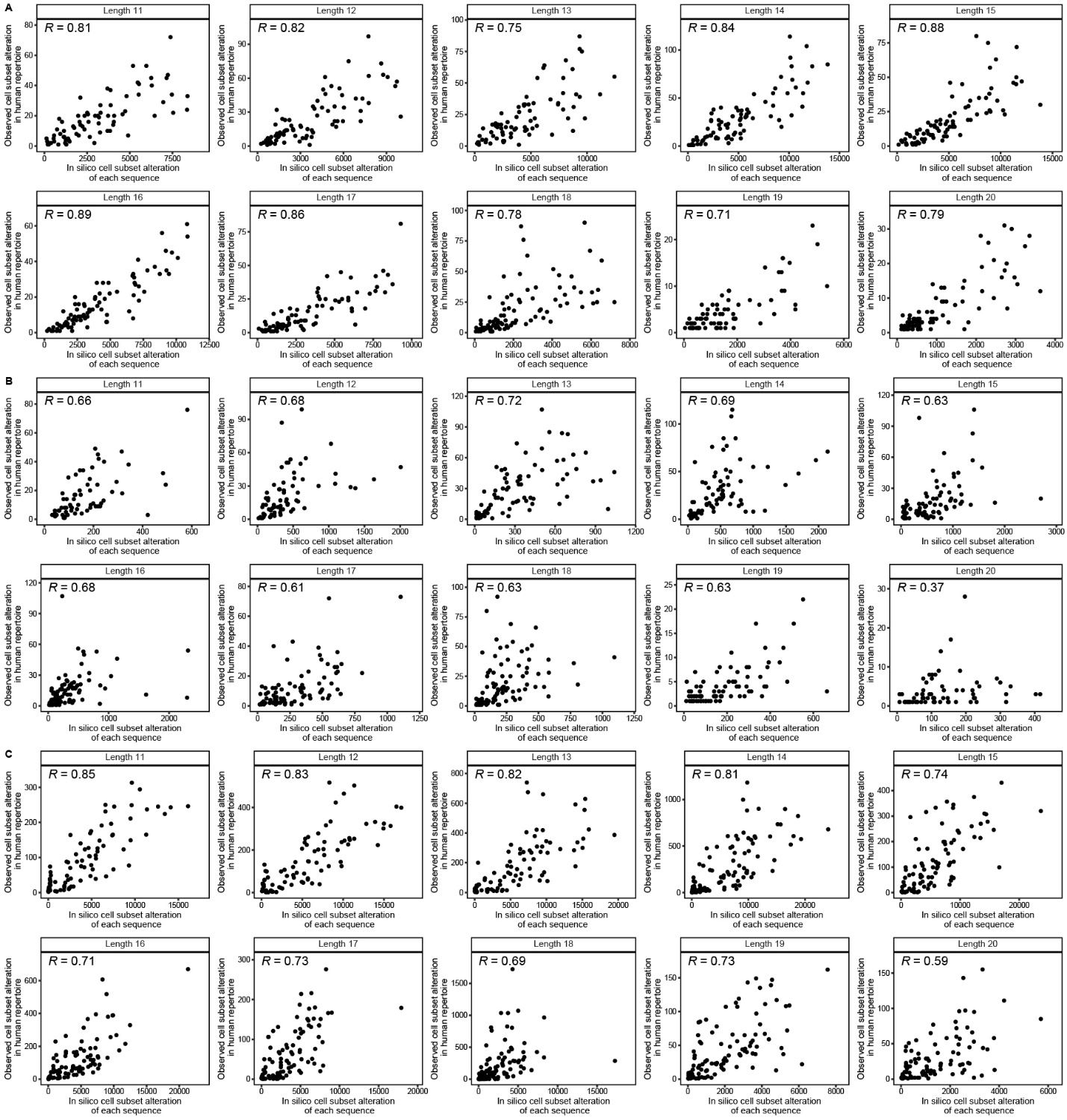


Supplementary Figure 2. Correlation plots between the cell subset alteration counts measured in *in silico* saturation mutagenesis and in human repertoire data. Alteration of B cell subset between naïve-memory (A), memory-ASC (B), and naïve-ASC (C) are shown, respectively. Lengths between 11 and 20, which comprise the majority of the dataset, are plotted. Spearman’s rank correlation coefficient is shown in the figure.


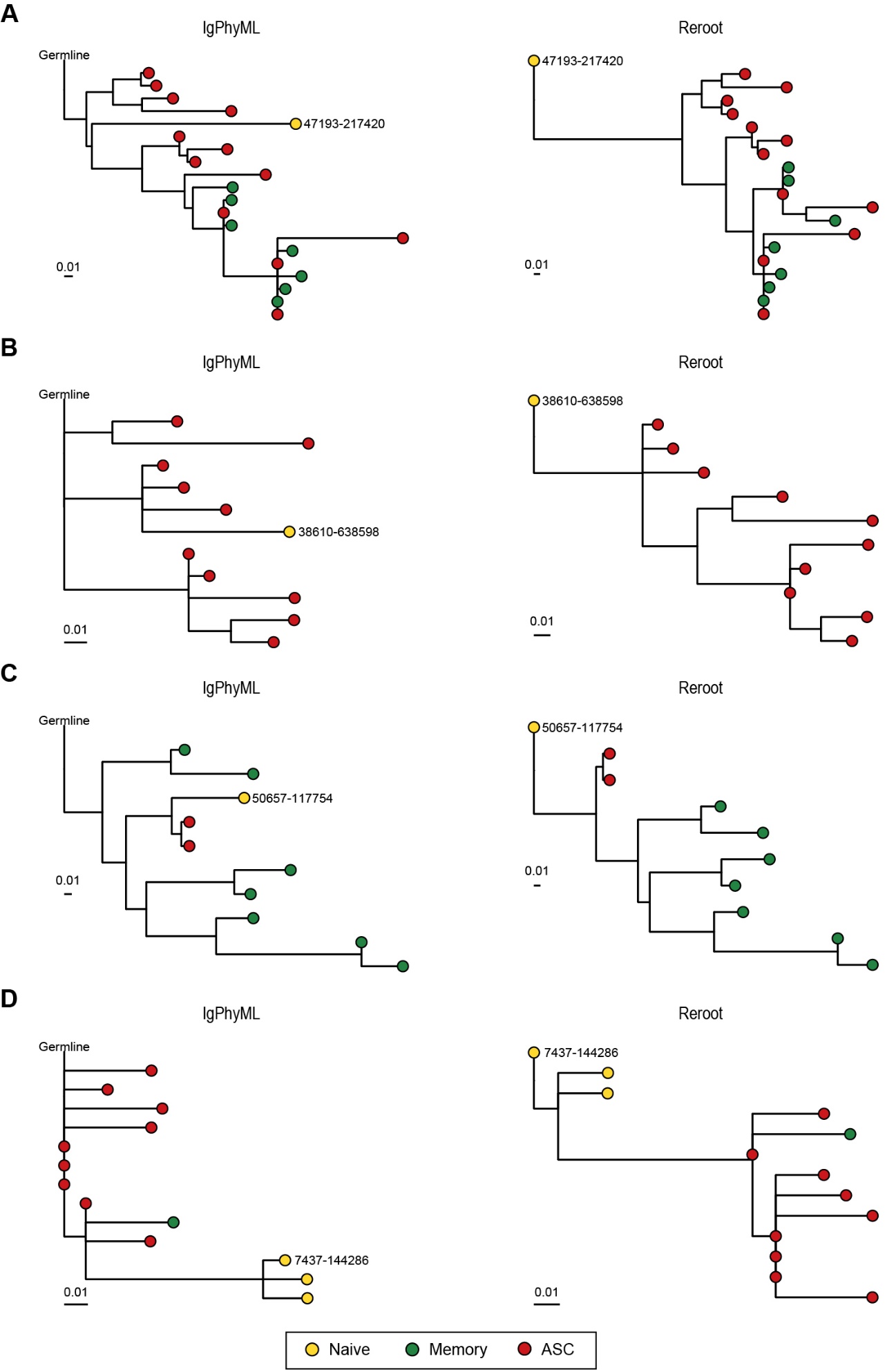


Supplementary Figure 3. Visualization of BCR phylogenetic trees reconstructed using IgPhyML and BCR-SORT. Examples of phylogenetic trees originating from various diseases are illustrated to compare the two different methods. The type of disease analyzed corresponds to influenza vaccination (A), tetanus toxoid vaccination (B), healthy (C), and systemic lupus erythematosus (D). Naïve B cell node selected as a new root of the tree is labeled.


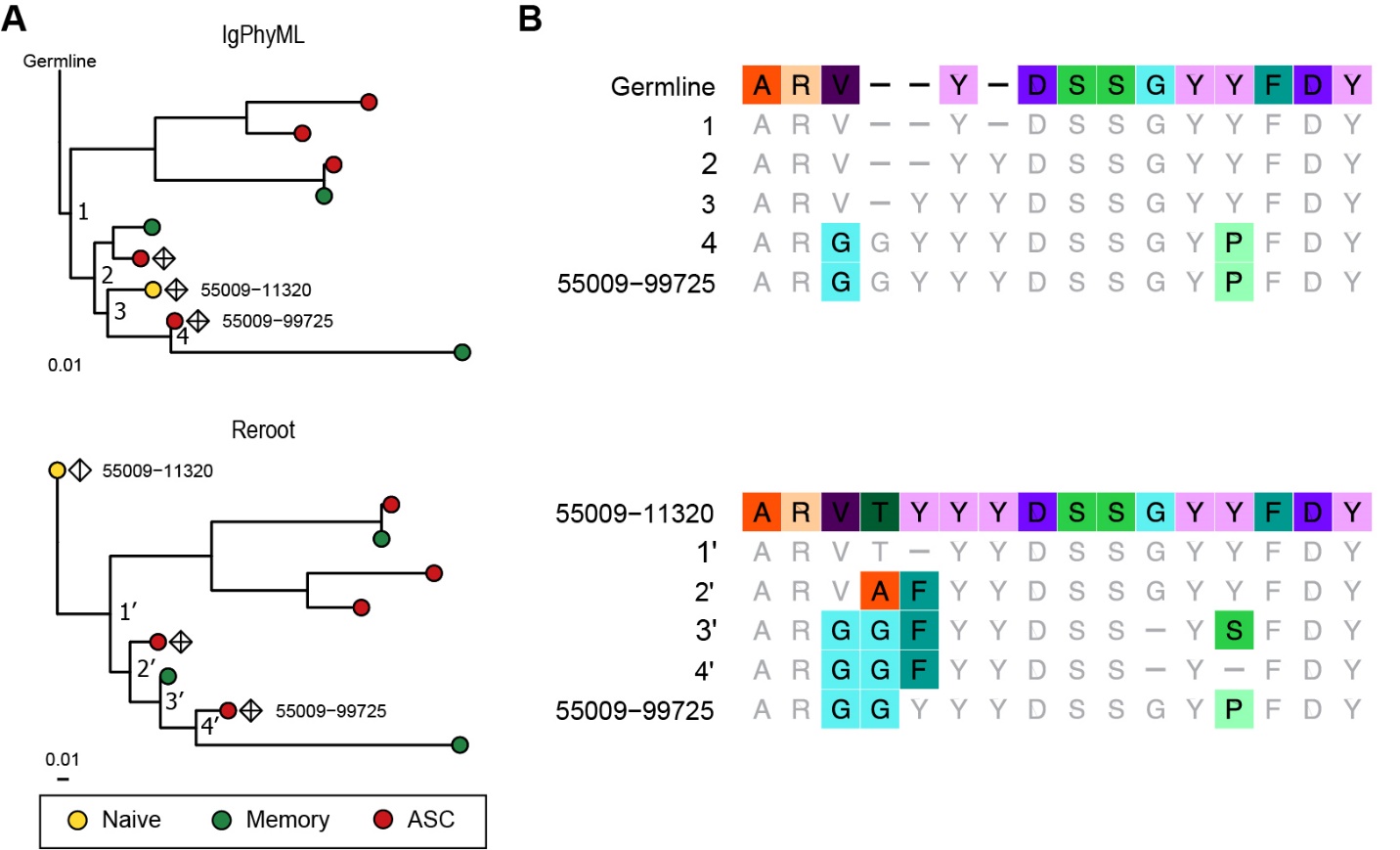


Supplementary Figure 4. Rearranged mutation history toward antigen-binding BCRs. (A) An example of phylogenetic tree representing the maturation toward antigen-specific sequences. BCR lineage from COVID-19 patient is reconstructed by IgPhyML (top) and rerooted using BCR-SORT (bottom). Verified antigen-specific BCRs are labeled as diamond. (B) Alignment of BCR sequences along the maturation path in a. Sequence variations along with the maturation path defined by IgPhyML (top) and rerooted using BCR-SORT (down) are shown. Compared with IgPhyML, rerooting using BCR-SORT yields clearer HCDR3 sequence variations from the root sequence to the binder sequence. Amino acids are color-labeled when mutated from the prior sequence.


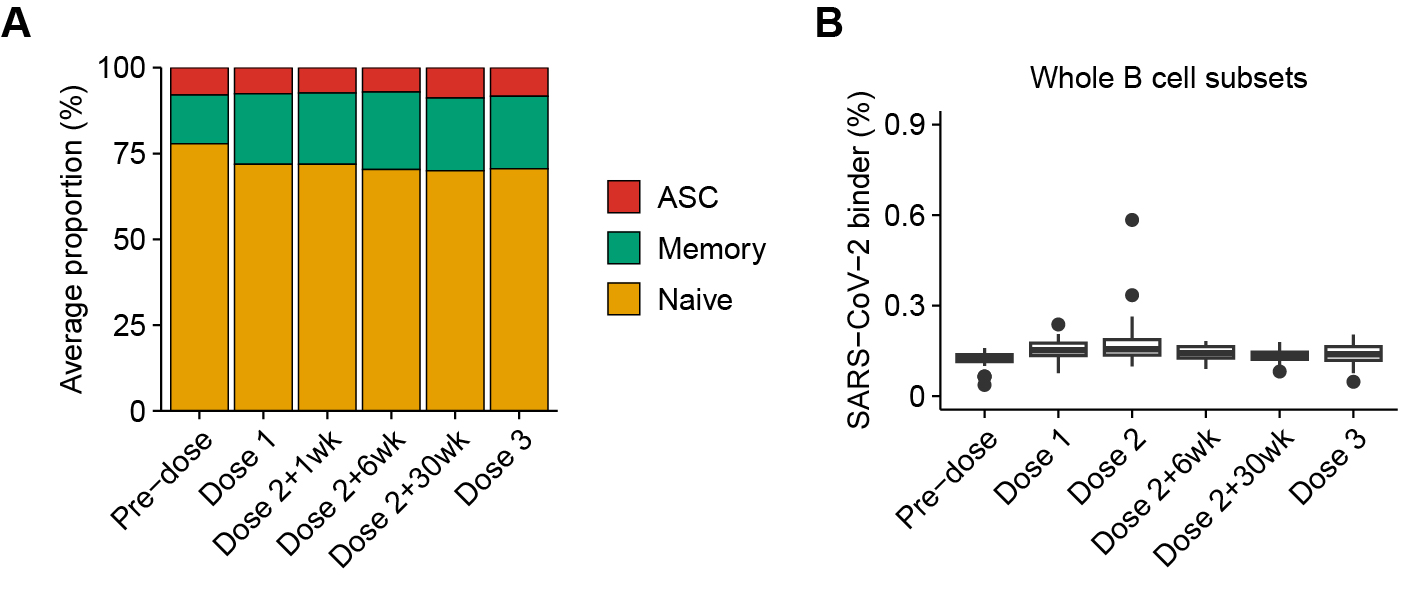


Supplementary Figure 5. Chronological variation of B cell subpopulations during triple COVID-19 vaccinations. BCR repertoires are identified at six different time points: pre-vaccination, 1 week after the first dose, 1, 6, 30 weeks after the 2^nd^ dose, and 1-4 weeks after the third dose. (A) Proportion of B cell subsets constituting the BCR repertoires predicted using BCR-SORT. Average values over the entire COVID-19 vaccine recipient (n=41) are shown. (B) Proportion of SARS-CoV-2-binding BCRs without using B cell subset predicted by BCR-SORT. Outliers lying outside 2 standard deviations from the mean are discarded for better visualization.


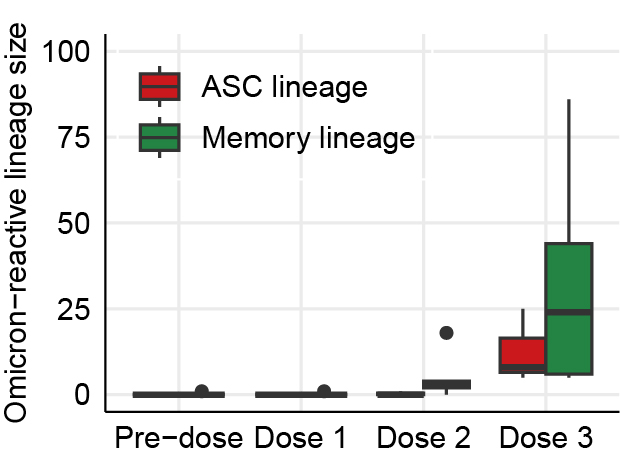


Supplementary Figure 6. Expansion of BCR lineages containing Omicron binders during consecutive vaccinations. Size distribution of lineages containing Omicron-binding BCRs after the 3^rd^ dose. Lineage size is defined as the number of unique BCRs, and those containing five or more BCRs are considered (Memory lineage; n = 5, ASC lineage; n = 3).


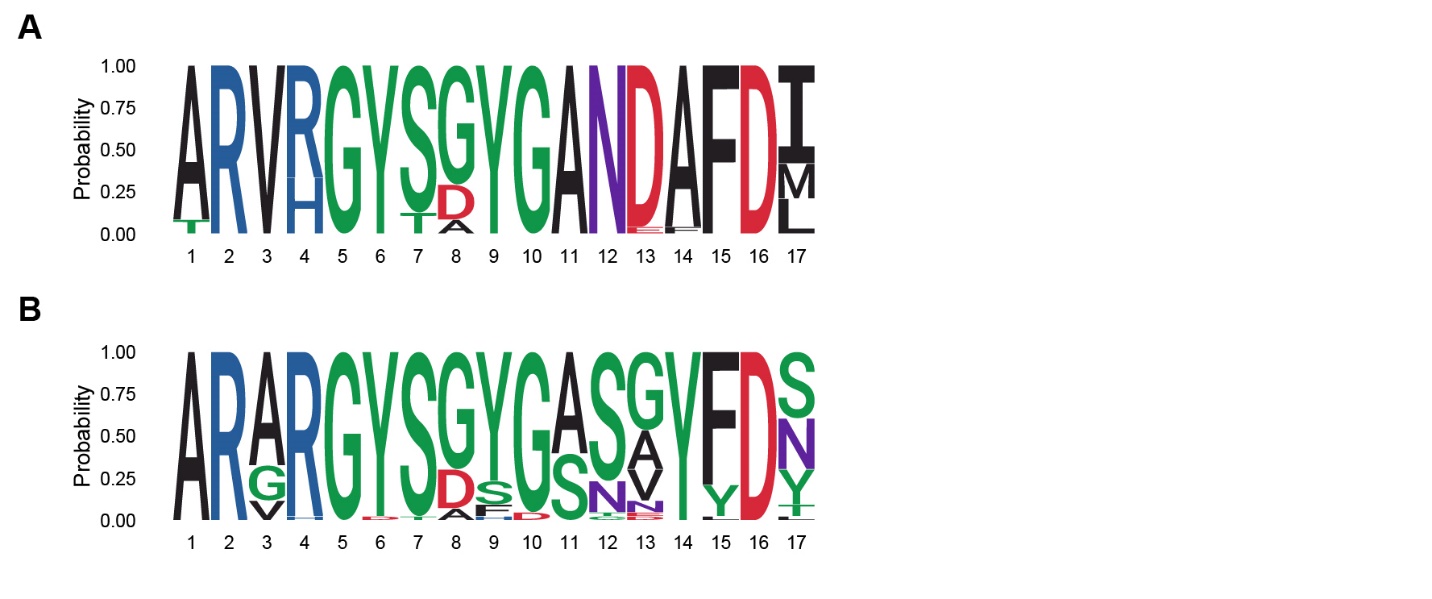


Supplementary Figure 7. Logo plots visualizing HCDR3 sequences within Omicron-specific lineages. Lineage #35-383691 (A) and #43-253663 (B) are shown, respectively.


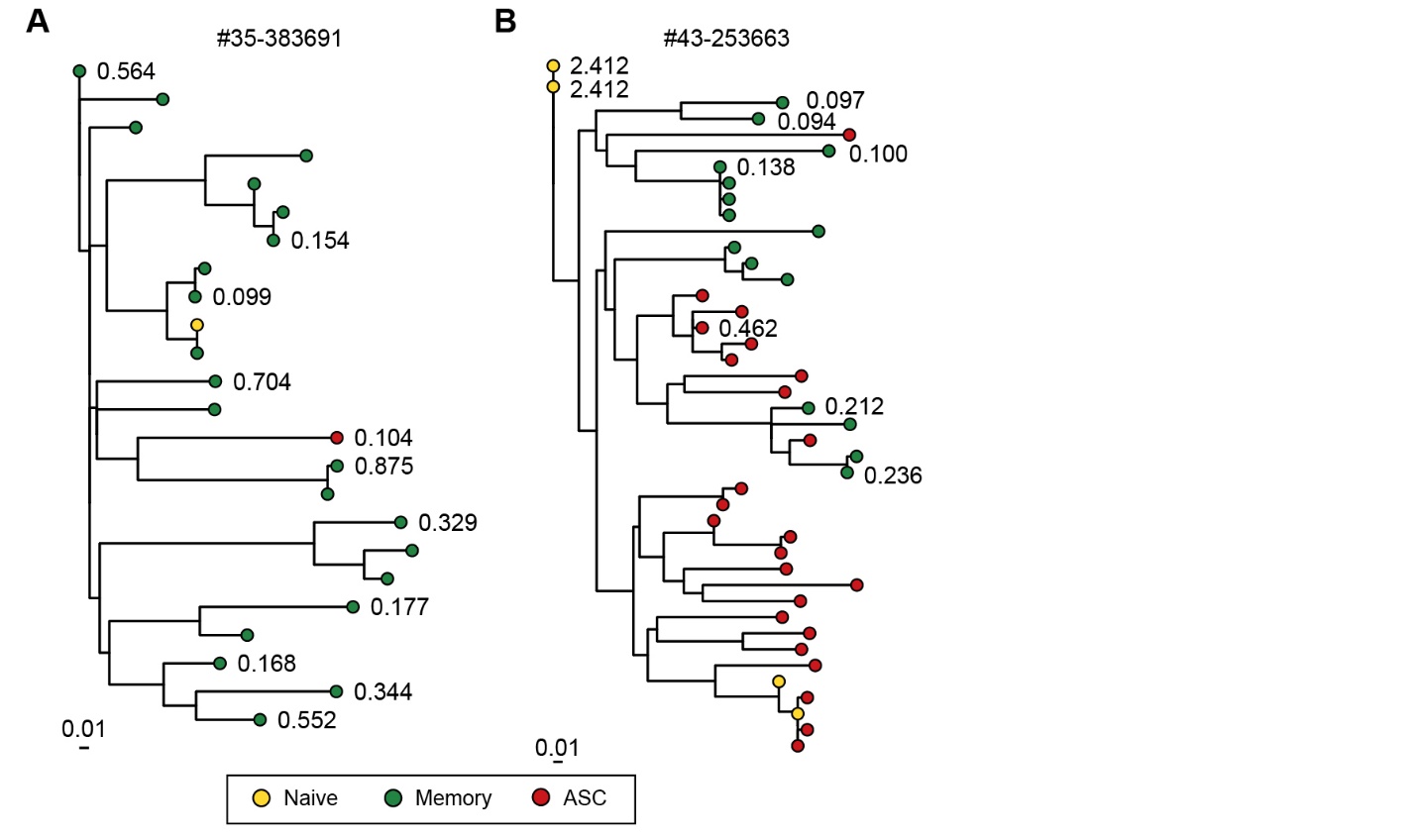


Supplementary Figure 8. Omicron reactivity of BCRs comprising the Omicron-binding memory B cell lineages. BCRs with verified EC50 (nM) values against Omicron viral proteins were shown for lineage #35-383691 (A) and #43-253663 (B), respectively.


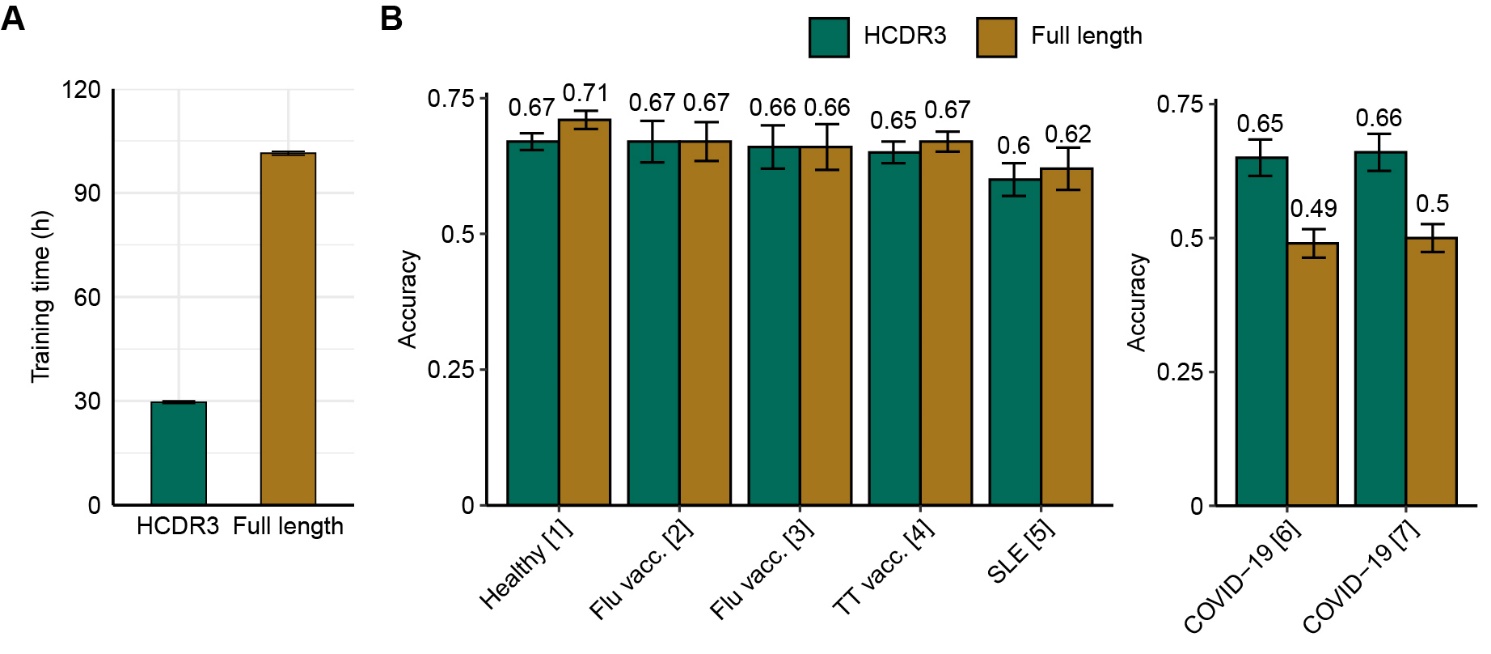


**Supplementary Figure 9. Comparison of HCDR3 and full-length model on benchmarking FACS and scRNA-seq.** Instead of using the HCDR3 sequence, the full-length BCR is employed as the input for BCR-SORT, and their performances are compared. (**A)** Training time of BCR-SORT. (**B)** Performance of BCR-SORT on external validation datasets, verified by FACS (left) and scRNA-seq (right). Owing to the misalignment between full-length BCR sequences generated by FACS and scRNA-seq, the performance of BCR-SORT deteriorates when applied to scRNA-seq data, indicating that requiring full-length BCR reduces the model’s applicability. Accuracy is measured using various benchmark datasets and presented according to the source of the dataset. Performance is evaluated on datasets for whom full-length nucleotide BCR sequences are available, and error bars of datasets containing only a single sample are calculated by bootstrapping 150 BCR sequences 1,000 times. Although the full-length BCR sequence offers more information as input, the increase in performance is marginal considering the substantial computational cost incurred.

**Supplementary Table 1. Isotype proportion of the dataset used for training.**

| **Label** | **IgM (%)** | **IgD (%)** | **IgG (%)** | **IgA (%)** | **IgE (%)** |
| --- | --- | --- | --- | --- | --- |
| Naive | 77.236 | 19.424 | 2.453 | 0.883 | 0.005 |
| Memory | 54.527 | 1.703 | 31.047 | 12.719 | 0.004 |
| ASC | 9.196 | 0.000 | 60.739 | 30.044 | 0.021 |

**Supplementary Table 2. Statistics of datasets used for benchmarking FACS and scRNA-seq.**

| **Benchmark** | **Name** | **Immunological condition** | **PMID** | **Sample** | **BCR count** |
| --- | --- | --- | --- | --- | --- |
| FACS | Healthy [1] | Healthy | 34661527 | 10 | 30,000 |
|  | Flu vacc. [2] | Influenza vaccination | 27525369 | 1 | 3,000 |
|  | Flu vacc. [3] | Influenza vaccination | 26006014 | 4 | 12,000 |
|  | TT vacc. [4] | Tetanus toxoid vaccination | 26006014 | 4 | 12,000 |
|  | SLE [5] | Systemic lupus erythematosus | 26006014 | 13 | 36,000 |
| scRNA-seq | COVID-19 [6] | COVID-19 infection | 33571429 | Pooled | 3,000 |
|  | COVID-19 [7] | COVID-19 vaccination | 35168246 | Pooled | 3,000 |
|  | Healthy [8] | Healthy | 33657410 | Pooled | 300 |
|  | COVID-19 [9] | COVID-19 infection | 33657410 | Pooled | 3,000 |
|  | COVID-19 [10] | COVID-19 infection | 32788748 | Pooled | 1,845 |
|  | COVID-19 [11] | COVID-19 infection | 33879890 | Pooled | 3,000 |

**Supplementary Table 3. Statistics of datasets used for training.**

| **Label** | **Immunological condition** | **PMID** | **Sample** | **BCR count** | **Sum** |
| --- | --- | --- | --- | --- | --- |
| Naive | Healthy/Celiac disease | 30733445 | 99 | 551498 |  |
| Naive | Influenza vaccination | 28179494 | 18 | 73297 |  |
| Naive | Healthy | 28179494 | 12 | 59470 |  |
| Naive | Healthy | 28959265 | 8 | 53994 |  |
| Naive | Healthy | 27005435 | 10 | 48000 |  |
| Naive | Tonsillitis/Obstructive-Sleep-Apnea | 33579751 | 8 | 36692 |  |
| Naive | Healthy | 28087666 | 4 | 30292 |  |
| Naive | Healthy | 25976772 | 5 | 20022 |  |
| Naive | Meningococcal vaccination | 25976772 | 4 | 16006 |  |
| Naive | Healthy | 27525369 | 3 | 7234 |  |
| Naive | Influenza vaccination | 27525369 | 2 | 6839 |  |
| Naive | Healthy | 27490633 | 1 | 3874 | 907218 |
| Memory | Healthy | 31767621 | 21 | 305000 |  |
| Memory | Tonsillitis/Obstructive-Sleep-Apnea | 33579751 | 8 | 178632 |  |
| Memory | Influenza vaccination | 27525369 | 14 | 156075 |  |
| Memory | Healthy | 27005435 | 10 | 92842 |  |
| Memory | Healthy | 25976772 | 5 | 50928 |  |
| Memory | Meningococcal vaccination | 25976772 | 4 | 40619 |  |
| Memory | Healthy | 27525369 | 3 | 27158 |  |
| Memory | Healthy | 28087666 | 4 | 23834 |  |
| Memory | Healthy | 27490633 | 2 | 13569 | 888657 |
| ASC | Influenza vaccination | 27849037 | 30 | 256942 |  |
| ASC | Meningococcal vaccination | 25976772 | 20 | 206752 |  |
| ASC | Healthy | 31767621 | 21 | 115087 |  |
| ASC | Tonsillitis/Obstructive-Sleep-Apnea | 33579751 | 8 | 99532 |  |
| ASC | IgG4-RD | 26006014 | 2 | 64913 |  |
| ASC | Influenza vaccination | 27525369 | 4 | 50509 |  |
| ASC | Ebola | 31104840 | 1 | 31199 |  |
| ASC | Pemphigus vulgaris | 26006014 | 2 | 30976 |  |
| ASC | Healthy | 27490633 | 2 | 5506 | 861416 |

**Supplementary Table 4. Ablation study on input attributes.**

| **Methods** | **Accuracy** |
| --- | --- |
| BCR-SORT | 0.833±0.002 |
| (w/o) CDR3 | 0.640±0.003 |
| (w/o) Isotype | 0.792±0.003 |
| (w/o) IGHV, IGHJ gene | 0.831±0.001 |
